# Serum Cystatin C Levels Could Predict Rapid Kidney Function Decline in A Community-Based Population

**DOI:** 10.3390/biomedicines10112789

**Published:** 2022-11-02

**Authors:** Wei-Ching Fang, Hsing-Yu Chen, Shao-Chi Chu, Po-Hsi Wang, Chin-Chan Lee, I-Wen Wu, Chiao-Yin Sun, Heng-Jung Hsu, Chun-Yu Chen, Yung-Chang Chen, Vin-Cent Wu, Heng-Chih Pan

**Affiliations:** 1 Department of Family Medicine, Linkou Chang Gung Memorial Hospital, Taoyuan 333, Taiwan; winds75526@gmail.com; 2 Graduate Institute of Clinical Medical Sciences, College of Medicine, Chang Gung University, Taoyuan 333, Taiwan; b8705016@gmail.com; 3 Division of Chinese Internal Medicine, Center for Traditional Chinese Medicine, Chang Gung Memorial Hospital, Taoyuan 333, Taiwan; 4 School of Traditional Chinese Medicine, College of Medicine, Chang Gung University, Taoyuan 333, Taiwan; 5 Division of Nephrology, Department of Internal Medicine, Keelung Chang Gung Memorial Hospital, Keelung 204, Taiwan; vicky11492@gmail.com (S.-C.C.); gray8741236@gmail.com (P.-H.W.); leefang@cgmh.org.tw (C.-C.L.); fliawu@yahoo.com (I.-W.W.); fish3970@gmail.com (C.-Y.S.); hsuaaron@gmail.com (H.-J.H.); shilewu@gmail.com (C.-Y.C.); 6 College of Medicine, Chang Gung University, Taoyuan 333, Taiwan; cyc2356@gmail.com; 7 Community Medicine Research Center, Keelung Chang Gung Memorial Hospital, Keelung 204, Taiwan; 8 Division of Nephrology, Department of Internal Medicine, Linkou Chang Gung Memorial Hospital, Taoyuan 333, Taiwan; 9 Division of Nephrology, Department of Internal Medicine, National Taiwan University Hospital, Taipei 100, Taiwan; q91421028@ntu.edu.tw; 10 Graduate Institute of Clinical Medicine, College of Medicine, National Taiwan University, Taipei 100, Taiwan

**Keywords:** biomarker, chronic kidney disease, cystatin C, rapid kidney function decline

## Abstract

Background: Several biomarkers have been correlated with the prevalence and severity of chronic kidney disease (CKD); however, the association between biomarkers and rapid kidney function decline (RKFD) is unknown. This study aimed to evaluate the predictive performance of biomarkers to determine who is likely to develop RKFD in a healthy population. Methods: A community-based cohort of 2608 people residing in northern Taiwan were enrolled, and their renal function was followed annually from January 2014 to December 2019. The outcomes of interest were RKFD, defined as a 15% decrease in the estimated glomerular filtration rate (eGFR) within the first 4 years, and a decrease in eGFR without improvement in the fifth year. Clinical variables and potential predictors of RKFD, namely adiponectin, leptin, tumor necrosis factor-alpha, and cystatin C, were measured and analyzed. Results: The incidence of RKFD was 17.0% (105/619). After matching for age and sex at a 1:1 ratio, a total of 200 subjects were included for analysis. The levels of cystatin C and total vitamin D were significantly negatively correlated with eGFR. eGFR was negatively correlated with the levels of cystatin C and total vitamin D. Among the biomarkers, cystatin C showed the best predictive performance for RKFD (area under the receiver operating characteristic curve: 0.789). Lower serum cystatin C was associated with a higher rate of RKFD in healthy subjects. A generalized additive model showed that 0.82 mg/L was an adequate cut-off value of cystatin C to predict RKFD. Multivariable logistic regression analysis further indicated that low cystatin C and eGFR were independent predictors of the possibility of RKFD. Conclusions: Serum cystatin C level could predict the possibility of RKFD. We suggest that a low cystatin C level should be considered as a risk factor for RKFD in healthy subjects.

## 1. Background

Chronic kidney disease (CKD) is a major global health issue. Due to the increasingly aging population and associated comorbidities, the incidence of CKD is increasing. The occurrence of CKD is associated with a higher risk of comorbidities such as cardiovascular events, stroke, infection, and peripheral vessel disease [1] as well as a higher mortality rate and higher medical expenses [2]. CKD is most commonly attributed to diabetes, hypertension, glomerulonephritis, infection, and exposure to nephrotoxic agents [3,4]. The early detection of CKD is important to prevent complications associated with this disease [5]. Furthermore, identifying healthy subjects with a higher risk of rapid kidney function decline (RKFD) is an even more important public health issue.

Several biomarkers have been correlated with the prevalence and severity of CKD, including metabolic syndrome markers (e.g., adiponectin and leptin) [6,7], inflammatory markers (e.g., tumor necrosis factor-alpha (TNF-α)) [8], mineral metabolism markers (e.g., total vitamin D) [9], and glomerular filtration markers (e.g., cystatin C) [5,10]. Metabolic syndrome is a strong independent risk factor for the development of cardiovascular disease (CVD) and CKD [11,12]. The increases in plasma adiponectin and leptin that occur when renal function deteriorates may represent an adaptive response to the altered metabolic profile and could be associated with the occurrence of CKD independently of traditional factors such as age, sex, smoking, alcohol intake, body mass index (BMI), diabetes, hypertension, and serum cholesterol. The role of inflammatory biomarkers in the etiology of CKD has also been well established. TNF-α is a key cytokine mediating both acute and chronic inflammation, and it has been shown to contribute to CKD independently of other established CKD risk factors, history of CVD, and the use of antihypertensive, oral hypoglycemia, and lipid-lowering agents. In addition, severe disturbances in mineral metabolism have been associated with the progression of CKD [13], and vitamin D has emerged as an important regulator of mineral homeostasis and biomarkers of early cardiovascular change [14]. Moreover, vitamin D deficiency has been associated with renal hyperfiltration (RHF), which may lead to overestimated renal function and further RKFD [15]. Cystatin C is a biomarker of glomerular filtration function. It does not seem to be influenced by race or muscle mass [16], and it has been reported to more accurately estimate the glomerular filtration rate than the serum creatinine concentration [17,18]. 

While previous studies have identified markers that may be associated with the prevalence and severity of CKD, few prognostic markers have been identified to distinguish healthy subjects who will experience RKFD from those who will experience a relatively slow decline in renal function. To the best of our knowledge, no prospective clinical study has investigated the relationship between the aforementioned biomarkers and RKFD in healthy individuals. The identification of potential associations may allow for risk surveillance and the targeted primary prevention of the occurrence of CKD. Therefore, the aim of this prospective study was to test for the independent associations of these biomarkers (adiponectin, leptin, TNF-α, total vitamin D, and cystatin C) with the risk of RKFD in a community-based cohort. 

## 2. Materials and Methods

### 2.1. Patient Information and Data Collection

This longitudinal, community-based cohort study was conducted from January 2014 to December 2019 in four districts of Northeastern Taiwan, namely Wanli, Anle, Ruifang, and Gongliao. A community outreach health screening program, including a physical examination, blood and urine laboratory tests, and a questionnaire survey, was performed to recruit the subjects. A standardized questionnaire was provided to all the participants by a trained team of interviewers to obtain information on their drinking, smoking, and betel nut chewing habits; exercise regime; medication history (oral hypoglycemic agents, insulin injections, statins, herbs, and hormones); family history; and physical and mental health status (Short Form Health Survey, sleeping quality survey, depression survey, and health knowledge). All of the participants agreed to sign the informed consent forms.

Basic physical measurements and laboratory data were recorded. Urine samples were collected to evaluate albuminuria and proteinuria, and the urine albumin creatinine ratio (UACR) and urine protein creatinine ratio (UPCR) were calculated. The presence of systemic disorders, such as hypertension, diabetes, CKD, and cardiovascular disease were recorded. We excluded the patients (1) who were lost to follow-up, (2) who declined to be enrolled in the study, or (3) who had undergone renal replacement therapy or organ transplantation before the study. Follow-up examinations were conducted after 1 year, during which the physical parameters, blood and urine laboratory test results, and the results of the survey were recorded again.

In total, 2608 completed the baseline survey and were invited to participate in this study. Of the 638 individuals who agreed to participate in the study, 619 successfully completed the 5-year annual follow-up, 13 died, and 6 were lost to follow-up.

### 2.2. CKD

CKD was defined as an eGFR of < 60 mL/min/1.73 m^2^, determined using the abbreviated Modification of Diet in Renal Disease equation or the presence of persistent proteinuria [19].

### 2.3. Metabolic Syndrome

Metabolic syndrome was defined as the presence of three out of five of the following criteria according to the National Cholesterol Education Program (NCEP) Adult Treatment Panel III (ATP III) Guidelines [20]:

(1) A waist circumference of ≥ 90 cm in men and ≥ 80 cm in women according to the modified Asian criteria.
(2) Triglycerides ≥ 150 mg/dL or treatment for elevated triglycerides.
(3) High-density lipoprotein cholesterol < 40 mg/dL in men or <50 mg/dL in women, or treatment for low high-density lipoprotein cholesterol.
(4) Blood pressure ≥ 130/85 mmHg or treatment for hypertension.
(5) Fasting glucose ≥ 100 mg/dL or previously diagnosed type 2 diabetes.

### 2.4. Homeostasis Model Assessment-Insulin Resistance (HOMA-IR) 

The HOMA-IR was calculated as fasting glucose (mg/dL) × fasting insulin (μIU/mL)/405 [21,22,23].

### 2.5. BMI 

BMI was calculated as the body weight divided by the square of the height (kg/m^2^).

### 2.6. Measurement of Serum Biomarker Levels

The concentrations of serum biomarkers were determined using enzyme-linked immunosorbent assay kits (adiponectin, Boster, Pleasanton, CA, United States; leptin, Boster, Pleasanton, CA, United States; high-sensitivity C-reactive protein (HS-CRP), Roche, Basel, Switzerland; TNF-α, Immunite 1000 LKNF1, Siemens Medical Solutions Diagnostics, Llanberis, UK) [24,25]. The serum level of 25(OH)D was measured using an electro-chemiluminescence immunoassay (Cobas® Vitamin D3 assay, Roche Diagnostics GmbH, Mannheim, Germany) with an interassay coefficient of variation of 2.2–13.6% [19]. Each biomarker assay was performed in duplicate according to the manufacturer’s instructions, and the mean value was used for further statistical analysis.

### 2.7. Outcome Assessment

All eligible participants were followed up for 5 years. The primary outcome of this study was RKFD, defined as a 15% decline in eGFR within the first 4 years and no improvement in eGFR decline in the fifth year [26], which was modified according to the definition from previous studies [27]. 

### 2.8. Statistical Analysis

Continuous variables were summarized as median and interquartile range (the distance between the first and third quartile). All variables were tested for normal distribution using the Kolmogorov–Smirnov test. Student’s t-test was used to compare the means of continuous variables and normally distributed data; otherwise, the Mann–Whitney U test was used. ANOVA was also used to compare the means of continuous variables and normally distributed data; otherwise, the Kruskal–Wallis test was used. Categorical data were tested using the chi-square test. Correlations of paired-group variables were assessed using linear regression and Pearson analyses. Discrimination was assessed using the area under the receiver operating characteristic curve (AUROC) values. An AUROC close to 0.5 indicates that the model performance approximates that of flipping a coin. The AUROC values were compared using a nonparametric approach. Net reclassification improvement (NRI) and integrated discrimination improvement (IDI) analyses were used to examine the role of potential biomarker and to stratify individuals into higher or lower risk categories (reclassification). We distinguished between risk categories (0.47) and reclassified the subjects with RKFD. A generalized additive model was plotted and adjusted for comorbidities, sex, and age in individual patients [28,29]. The model incorporated subject-specific random effects, expressed as the logarithm of the odd (logit), and the optimal cutoff value was defined as a log odds value of zero [30]. The variables were assessed by multivariable analysis using a multiple logistic regression model based on the forward elimination of data. Kaplan–Meier curves were used to estimate the survival function of time and were compared with the log-rank test. Furthermore, subgroup analyses for RKFD were performed, including age (>60 and ≤60 years), sex, and co-morbidities (hypertension, diabetes, metabolic syndrome, CVD, and gout), and interactions between cystatin C and the covariates were also examined. To validate the study results, sensitivity tests with different propensity score (PS) models were performed [31]. For different PS models, the inverse probability of treatment weighting (IPTW), propensity score matching (PSM), and 5-block stratification were used [32]. All statistical tests were two-tailed; a value of *p* < 0.05 was considered statistically significant. Data were analyzed using SPSS (22.0, SPSS Inc., Chicago, IL, USA) and Stata software (16.0, StataCorp LLC, College Station, TX, USA) for Windows 10.

## 3. Results

### 3.1. Characteristics of the Study Subjects

**Figure 1:**
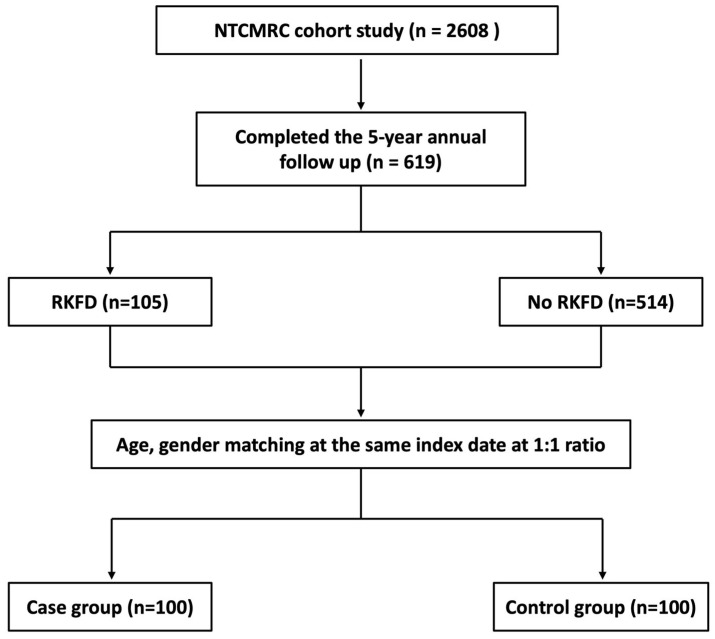
Flow diagram of this study. Abbreviations: NTCMRC, Northeastern Taiwan Community Medicine Research Cohort; RKFD, rapid kidney function decline.

Among the 619 subjects, 105 (17.0%) developed RKFD during the 5-year study period. To determine the correlations between the biomarkers (adiponectin, leptin, TNF-α, cystatin C, and total vitamin D) and RKFD, we further matched the subjects by age and sex at the same index date at a 1:1 ratio. The study flowchart is shown in Figure 1.

A total of 200 subjects were included for further analysis, with 100 in the group with RKFD and 100 in the group without RKFD. The baseline characteristics of the two groups are shown in Table 1. The median age of the subjects was 60.0 years, and 45 subjects were men (22.5%). The RKFD group had a higher prevalence of metabolic syndrome and higher levels of eGFR and HOMA-IR but lower levels of creatinine, total cholesterol, and LDL than the group without RKFD. With regard to the biomarkers, the RKFD group had a lower cystatin C level. The levels of adiponectin, leptin, TNF-α, and total vitamin-D were similar between the groups with and without RKFD. With regard to medications, the usage rates of oral hypoglycemic agents, anti-hypertensives, and painkillers were similar between the two groups.

We also compared the social psychology variables of the study population (Supplementary Table S1). The two groups had similar education levels, substance use practices, and dietary habits.

### 3.2. Cystatin C Could Predict RKFD in Healthy Population

**Figure 2:**
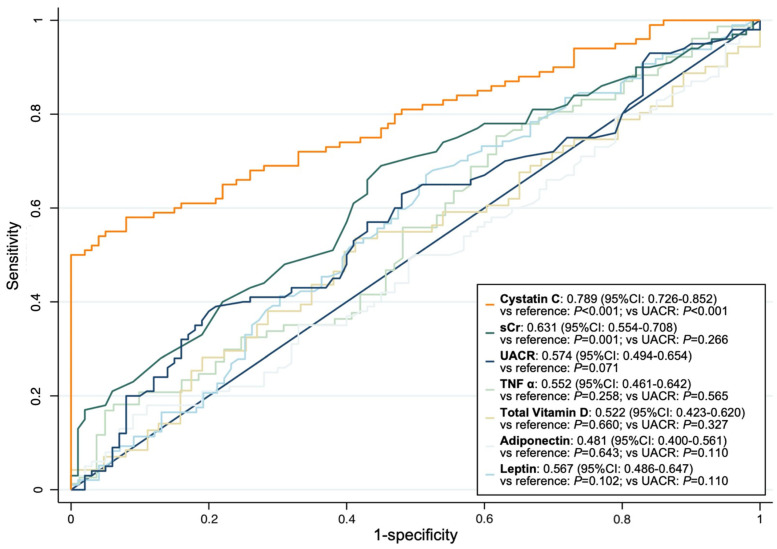
The area under the receiver operating characteristic curve of cystatin C, UACR, and sCr to predict the occurrence of 5-year RKFD. Abbreviations: RKFD, rapid kidney function decline; sCr, serum creatinine; TNF-α, tumor necrosis factor-α; UACR, urine albumin-to-creatinine ratio.

**Figure 3:**
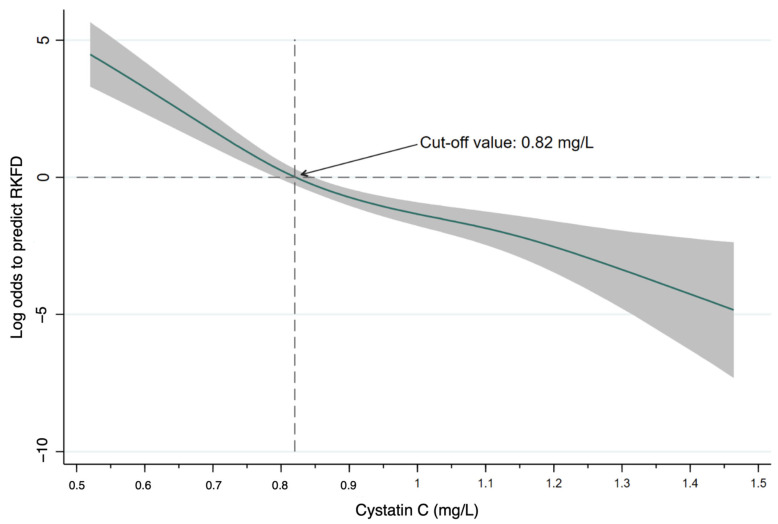
Generalized additive model plot for the probability of RKFD for cystatin C. Abbreviations: RKFD, rapid kidney function decline.

We examined the correlations between eGFR, UACR, and the serum levels of biomarkers at the baseline of the study (Table 2). eGFR was significantly negatively correlated with the levels of cystatin C and total vitamin D, while serum creatinine was significantly positively correlated with the levels of cystatin C and total vitamin D and negatively correlated with the level of leptin. The level of cystatin C was also significantly positively correlated with the levels of vitamin D and adiponectin. Based on AUROC analysis, cystatin C had the best discriminatory power for RKFD (AUROC: 0.789, 95% CI: 0.726–0.852) (Figure 2). We used a nonlinear generalized additive model to identify adequate cut-off values of continuous parameters to predict RKFD (Figure 3). All of the relevant covariates, including the baseline characteristics, comorbidities, and laboratory data listed in Table 1 were included. The results showed that a lower cystatin C level (cut-off value: 0.82 mg/L) was associated with a higher possibility of RKFD. We further divided the patients into groups with high (>0.82 mg/L) and low (≤0.82 mg/L) cystatin C levels (Table 3). The incidence of RKFD in the group with low cystatin C was significantly higher than that in the group with high cystatin C (*p* < 0.001). In addition, the group with low cystatin C was significantly younger (*p* < 0.001) and had a lower proportion of male subjects (*p* = 0.017). Most comorbidities were not significantly different between the groups with high and low levels cystatin C (*p* > 0.05). The prevalence rates of hypertension (*p* = 0.007) and the use of anti-hypertensives (*p* = 0.003) in the group with low cystatin C were significantly lower than those in the group with high cystatin C. The group with low cystatin C had significantly lower levels of blood urea nitrogen (*p* = 0.007), creatinine (*p* < 0.001), and uric acid (*p* = 0.018) but higher eGFR (*p* < 0.001) and albumin (*p* < 0.001) levels than the group with high cystatin C. With regard to the biomarkers, the low cystatin C group had a significantly lower total vitamin D (*p* = 0.028) and adiponectin (*p* = 0.002) levels. The levels of leptin and TNF-α were similar between the groups with low and high cystatin C levels.

### 3.3. Analysis of Factors Associated with the Possibility of RKFD

**Figure 4:**
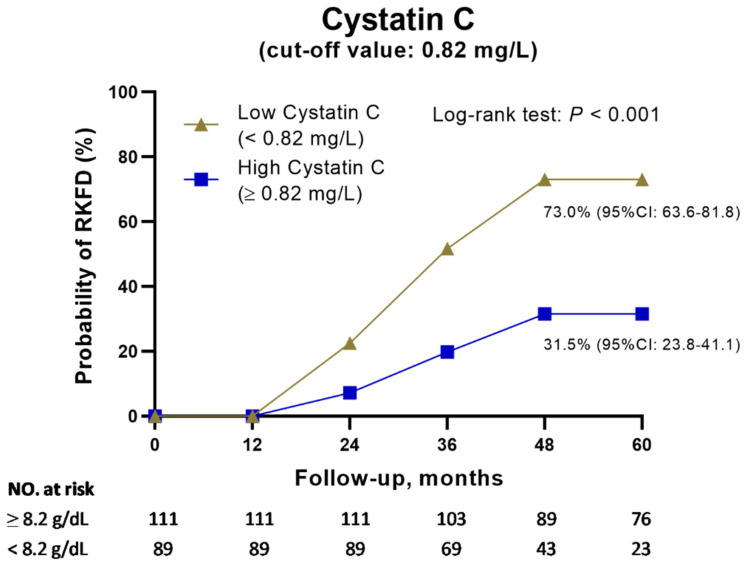
Kaplan–Meier curves for the probability of the occurrence of RFKD stratified by cystatin C. Abbreviation: RKFD, rapid kidney function decline.

Figure 4 illustrates stratified cumulative probabilities of the occurrence of RFKD according to cystatin C level and demonstrates that the group with low cystatin C had a significantly higher cumulative RKFD rate than the group with high cystatin C (low- vs. high-cystatin C group = 73.0% vs. 31.5%, *p* < 0.001). Multivariable logistic regression analysis showed that low cystatin C (OR: 20.35, 95% CI: 6.44–64.29) and eGFR (OR: 1.40, 95% CI: 1.06–1.85) had independent prognostic significance for the possibility of RKFD (Table 4). Furthermore, NRI and IDI analyses were used to distinguish risk categories (0.47) and to reclassify the subjects with RKFD into high- and low-risk categories (Supplementary Figure S1; Supplementary Table S2). Incorporating cystatin C with base covariates led to a significant increase in risk stratification (categorical NRI = 0.29; standard error: 0.0943; *p* = 0.002). Most of this effect came from the subjects without RKFD (event IDI = 0.26; standard error: 0.031; *p* < 0.001). Of note, the subjects with a low cystatin C level had a significantly higher eGFR than those with a high cystatin C level at all time points. However, they had a greater reduction in eGFR during the study period (low- vs. high-cystatin C group = 18.73% vs. 14.77%, *p* < 0.001) (Supplementary Figure S2). 

### 3.4. Subgroup and Sensitivity Analyses

Supplementary Table S3 shows the results of the subgroup analysis. The trends of the risk for RKFD were similar among all subpopulations, and interactions between cystatin C ≥ 0.82 mg/L and stratified covariates were found. The association between a low cystatin C level and a higher risk of RKFD was more significant in the patients who were younger, male, and had cardiovascular syndrome (*p* < 0.001) as well as in those without hypertension, diabetes, metabolic syndrome, or gout (*p* < 0.001). Supplementary Table S4 shows the results evaluated using different models, including eligible cases with different PS modeling. The PS models were generated by predicting the occurrence of low cystatin C (≤0.82 mg/L) using the aforementioned demographic features, including sex, comorbidities (hypertension, diabetes, metabolic syndrome, CVD, and gout), and medications (oral hypoglycemic agents, antihypertensives, and painkillers). In the IPTW model, 1/PS represented the weight of patients with low cystatin C levels, while 1/1-PS represented patients with high cystatin C levels [33]. For PSM, the PS was used to obtain a similar number of patients with low cystatin C and high cystatin C levels at a 1:1 ratio, with the caliper set to 0.1 [32]. The association between a low cystatin C level and a higher risk of RKFD was similar in all models.

## 4. Discussion

In this community-based study, the incidence of RKFD was 17.0% (105/619), which is consistent with previous studies [34]. Clinical tools for assessing the risk of RKFD remain limited. To the best of our knowledge, this study is the first to evaluate the predictive performance of biomarkers for RKFD in a healthy population. Our results demonstrated that a low level of serum cystatin C (<0.82 mg/L) was associated with a higher risk of developing RKFD in this healthy population. Of the analyzed potential biomarkers, cystatin C was the strongest predictor of RKFD. In addition, multivariable logistic regression indicated that serum cystatin C level and eGFR were independent predictors of the occurrence of RKFD. Moreover, the independent association between a low cystatin C level and high risk of RKFD was consistent across subgroups of age, sex, CVD, hypertension, diabetes, metabolic syndrome, and gout, and these results remained robust in the sensitivity analyses. 

Cystatin C is a 13 kDa protein produced by nucleated cells at a steady rate. It is freely filtered by glomerular cells and then reabsorbed and catabolized in proximal tubules [16]. Over the past few decades, it has been used as a filtration biomarker to evaluate kidney function, and it is currently available clinically [17]. Previous investigations have demonstrated that cystatin C has good diagnostic accuracy for acute kidney injury in patients undergoing cardiac surgery and that it is a good predictor of the risk of death and cardiovascular events in elderly persons [35,36]. However, clearly defined cutoff values of cystatin C have yet to be determined in different clinical settings, and this has limited its utility in clinical practice. In the current study, we demonstrated the predictive performance of cystatin C for RKFD in a healthy population and that a cystatin C level < 0.82 mg/L could predict the likelihood of RKFD. During the study period, the subjects with a low cystatin C level (<0.82 mg/L) had a significantly higher rate of eGFR decline than those with a high cystatin C level (≥0.82 mg/L) (low vs. high cystatin C: 18.73% vs. 14.77%, *p* < 0.001). Notably, the subjects with a low cystatin C level had significantly lower baseline levels of blood urea nitrogen, serum creatinine, and uric acid as well as higher baseline eGFR and albumin levels than those with a high cystatin C level, suggesting that the predictive ability of cystatin C was independent of initial kidney function and traditional renal function markers. 

Intraglomerular hypertension impacts the selectivity of the glomerular capillary barrier and leads to glomerular damage [37]. Previous studies have reported that RHF can lead to the onset of proteinuria and CKD in patients with diabetes mellitus [38,39]. In this study, our model suggested an association between higher baseline eGFR values and RKFD in this community-based population, supporting the role of aggravated kidney hyperfiltration on RKFD in healthy populations. In the literature, an eGFR ≥ 120 mL/min/1.73 m^2^ is regarded as RHF in the elderly [40]. The prevalence of RHF was 15.2% (94/619) in our primary cohort before age- and sex-matching. Given the high prevalence of RHF, more aggressive prevention and strict practices for associated risk factors, including hyperglycemia, hypertension, obesity, and smoking [38,39,40,41,42], might also help to prevent further RKFD for these patients. Additional well-powered research is needed to study this issue.

The mechanisms of RKFD are complex and multifactorial. Metabolic syndrome, chronic inflammation, and disturbances in mineral metabolism and glomerular hyperfiltration have been associated with the occurrence of CKD [8,10,11,12,13]. As shown in this study, the subjects with a low cystatin C level had significantly lower levels of adiponectin and vitamin D than those with a high cystatin C level. Previous studies have reported a positive correlation between adiponectin and cystatin C in patients with type 2 diabetes [43]. Reduced adiponectin levels are associated with reduced fatty acid oxidation, upregulated gluconeogenesis, and increased insulin resistance [44]. Low serum adiponectin levels are thought to be associated with the occurrence of diabetes and metabolic syndrome [45]. In our study, subjects with cystatin C < 0.82 mg/L tended to experience higher HOMA-IR increases than those with cystatin C ≥ 0.82 mg/L (low- vs. high-cystatin C group: 0.71 vs. 0.63). Although the difference was not statistically significant, this finding suggests that cystatin C levels could be correlated, but it remains to be determined whether the occurrence of low levels of vitamin D in individuals low cystatin C levels is correlated to altered mineral metabolism. The association of cystatin C with adiponectin and vitamin D signifies the potential of cystatin C to reflect multiple mechanisms for RKFD, which might explain, at least in part, why cystatin C, but not other biomarkers, was an independent predictor of the risk of RKFD. 

UACR is an important predictor for the onset and progression of early CKD, especially in patients with diabetes [46,47]. In this study, the prevalence of diabetes was low, and no subjects experienced significant proteinuria (UACR ≥ 30 mg/g). The poor predictive performance of UACR for RKFD may be due to the low UACR levels in this healthy population. In contrast, cystatin C had good predictive performance for RKFD in the healthy population. In the subgroup analysis, we found that the trend of the association between a low cystatin C level and RKFD was more significant in the younger subjects and in the subjects without diabetes, hypertension, metabolic syndrome, and gout. Considering that aging, diabetes, hypertension, metabolic syndrome, and uric nephropathy are major etiologies of CKD [11,12,48,49], cystatin C appears to have important clinical implications for assessing the risk of RKFD in healthy subjects. These findings support our hypothesis that incorporating biomarkers into clinical practice may improve clinical decision making when screening subjects who are at risk of RKFD. The early identification of subjects who are at risk of RKFD may allow for timely and targeted interventions.

In spite of the encouraging results observed in this study, several potential limitations should be recognized. First, the fact that our study involved patients of the same ethnicity limits the generalizability of the findings to other regions with different ethnic populations. Second, we lacked detailed information on drug use and dietary changes over time, so we were unable to study the effects of RAAS blockers and SGLT inhibitors on RKFD and the effect of dietary changes on eGFR calculations. However, our logistic regression model indicated that the use of oral hypoglycemic agents, anti-hypertensives, and baseline diet habits were not independent predictors for RKFD. Third, the predictive value of cystatin C for the risk of mortality has been well documented in many clinical scenarios [50,51]. However, the serum cystatin C level was not measured sequentially in this study, and the role of sequential measurements of serum cystatin C with the highest and lowest values may be more useful predictive markers than the initial serum cystatin C level alone. However, the timing of the occurrence of the highest and lowest serum cystatin C level is not specific, so it is difficult to apply clinically. Fourth, most of the participants were female (77.5%). Fifth, there is still the possibility of unmeasured confounding factors. Sixth, the predictive accuracy of logistic regression models has its own limitations. Finally, we also acknowledge that the observational nature of the study without a pre-specified protocol for the intervention cannot conclude causal relationships. Therefore, we can only speculate that serum cystatin C level may be a prognostic variable, and further studies are needed to validate our results. 

## 5. Conclusions

Our results showed that a serum cystatin C level < 0.82 mg/L could be considered as an independent risk factor for RKFD. We suggest that the serum cystatin C level is accurate and capable of assessing the risk of RKFD in healthy subjects.

## Figures and Tables

**Table 1:** Baseline characteristics of the study population.

	Total (*n* = 200)	RKFD (*n* = 100)	No RKFD (*n* = 100)	*p*
Demographics				
Age, years	60.00 (52.00–67.00)	58.00 (51.50–64.50)	60.50 (53.00–68.00)	0.19
Male gender, *n*	45 (22.5%)	20 (20.0%)	25 (25.0%)	0.40
Hypertension, *n*	50 (25.0%)	29 (29.0%)	21 (21.0%)	0.19
DM, *n*	24 (12.0%)	15 (15.0%)	9 (9.0%)	0.19
CKD, *n*	4 (2.0%)	2 (2.0%)	2 (2.0%)	1.00
Cardiovascular disease, *n*	14 (7.0%)	8 (8.0%)	6 (6.0%)	0.58
CVA, *n*	3 (1.5%)	3 (3.0%)	0 (0.0%)	0.081
HBV, *n*	25 (12.5%)	13 (13.0%)	12 (12.0%)	0.83
HCV, *n*	5 (2.5%)	2 (2.0%)	3 (3.0%)	0.65
Gout, *n*	7 (3.5%)	5 (5.0%)	2 (2.0%)	0.25
Autoimmune disease, *n*	2 (1.0%)	2 (2.0%)	0 (0.0%)	0.16
Metabolic syndrome	59 (29.5%)	38 (38.0%)	21 (21.0%)	**0.008**
Biochemical and physiological profiles				
SBP, mmHg	131.00 (119.00–141.00)	128.50 (118.00–142.50)	131.00 (120.50–141.00)	0.55
BMI, kg/m^2^	23.98 (22.23–26.35)	24.16 (22.22–26.23)	23.87 (22.25–26.87)	0.83
Central obesity, *n*	86 (43.0%)	48 (48.0%)	38 (38.0%)	0.15
Hgb, g/dL	13.70 (12.90–14.60)	13.80 (12.65–14.60)	13.70 (13.00–14.60)	0.78
Total cholesterol, mg/dL	208.50 (191.00–230.50)	199.50 (187.50–223.50)	215.00 (196.00–234.50)	**0.005**
LDL cholesterol, mg/dL	123.15 (105.85–146.10)	117.80 (101.15–140.15)	131.95 (112.20–152.40)	**0.010**
HDL cholesterol, mg/dL	56.55 (47.60–66.95)	54.70 (46.15–65.05)	57.50 (49.20–71.60)	0.13
Triglyceride, mg/dL	95.50 (69.00–139.00)	93.00 (67.50–156.50)	96.50 (69.50–130.50)	0.57
BUN, mg/dL	12.00 (10.00–15.00)	12.00 (10.00–15.00)	13.00 (11.00–15.00)	0.31
Creatinine, mg/dL	0.63 (0.55–0.76)	0.60 (0.54–0.70)	0.68 (0.57–0.81)	**0.001**
eGFR, ml/min/1.73 m^2^	101.27 (87.33–115.08)	106.77 (91.65–123.41)	96.24 (82.77–107.93)	**<0.001**
Uric acid, mg/dL	5.10 (4.30–6.10)	5.00 (4.20–6.00)	5.15 (4.50–6.20)	0.38
Albumin, g/dL	4.70 (4.50–4.90)	4.70 (4.50–4.90)	4.70 (4.50–4.80)	0.26
GPT, U/L	22.00 (17.00–29.50)	22.00 (17.00–31.00)	21.00 (17.00–28.00)	0.37
UACR, mg/g	5.75 (3.85–9.00)	6.35 (3.90–10.55)	5.20 (3.85–7.95)	0.071
Fasting glucose, mg/dL	96.00 (91.50–104.00)	97.00 (92.00–108.00)	95.50 (91.00–102.00)	0.19
HbA1C, %	5.60 (5.40–6.00)	5.60 (5.40–6.05)	5.60 (5.40–5.95)	0.81
Insulin, μIU/ml	5.80 (4.06–9.30)	6.00 (4.68–9.96)	5.20 (3.67–8.82)	0.060
HOMA-IR	1.43 (0.92–2.42)	1.51 (1.18–2.62)	1.31 (0.85–2.09)	**0.026**
HS-CRP, mg/L	0.94 (0.41–2.12)	0.94 (0.40–2.05)	0.91 (0.42–2.21)	0.54
Biomarkers				
Adiponectin, ng/ml	5.56 (3.31–10.14)	5.37 (3.17–9.84)	5.56 (3.52–10.35)	0.64
Leptin, ng/mL	12.00 (7.80–17.70)	12.90 (8.80–18.40)	10.90 (7.00–17.30)	0.11
Cystatin C, mg/L	0.86 (0.75–1.02)	0.75 (0.60–0.90)	0.93 (0.83–1.10)	**<0.001**
TNF-α	6.73 (5.70–8.17)	6.76 (6.14–8.32)	6.62 (5.31–7.97)	0.26
Total Vitamin D	24.29 (19.55–31.45)	25.25 (19.16–33.07)	24.19 (19.55–31.29)	0.66
Medication use				
OHAs, *n*	22 (11.1%)	15 (15.3%)	7 (7.0%)	0.063
Anti-hypertensives, *n*	46 (23.4%)	27 (27.8%)	19 (19.0%)	0.14
Painkillers, *n*	28 (14.8%)	18 (19.6%)	10 (10.3%)	0.073

Abbreviations: BMI, body mass index; BP, blood pressure; BUN, blood urea nitrogen; CVA, cerebrovascular accident; DM, diabetes mellitus; eGFR, estimated glomerular filtration rate; GPT, glutamic pyruvic transaminase; HbA1C, glycated hemoglobin; HOMA-IR, homeostatic model assessment-insulin resistance; HDL, high-density lipoprotein; HS-CRP, high-sensitivity C-reactive protein; LDL, low-density lipoprotein; OHA, oral hypoglycemic agents; RKFD, rapid kidney function decline; TNF-α, tumor necrosis factor-α; UACR, urine albumin-to-creatinine ratio. Values in bold are statistically significant (*p* < 0.05).

**Table 2:** Correlation between eGFR, UACR, and the serum levels of biomarkers at baseline (Pearson correlation coefficients: r).

	eGFR	Creatinine	UACR	Cystatin C	Leptin	Adiponectin	Total Vit. D	TNF-α
eGFR	-	−0.799 ***	0.064	−0.488 ***	0.014	−0.105	−0.246 **	0.088
Creatine	−0.799 ***	-	−0.122	0.392 ***	−0.153 *	−0.055	0.378 ***	−0.016
UACR	0.064	−0.122	-	0.010	0.061	−0.005	0.221 *	−0.047
Cystatin C	−0.488 ***	0.392 ***	0.010	-	−0.013	0.173 *	0.173 *	0.087
Leptin	0.014	−0.153 *	0.061	−0.013	-	−0.061	−0.053	0.052
Adiponectin	−0.105	−0.055	−0.005	0.173 *	−0.061	-	−0.014	−0.082
Total Vit. D	−0.246 **	0.378 ***	0.221 *	0.173 *	−0.053	−0.014	-	0.138
TNF-α	0.088	−0.016	−0.047	0.087	0.052	−0.082	0.138	-

Abbreviations: eGFR, estimated glomerular filtration rate; UACR, urine albumin-to-creatinine ratio; Vit. D, vitamin D. * *p* < 0.05. ** *p* < 0.01. *** *p* < 0.001.

**Table 3:** Demographic characteristics of the enrolled subjects stratified by a cut-off value of cystatin C of 0.82 mg/L.

	Total (*n* = 200)	Low Cystatin C (<0.82 mg/L) (*n* = 89)	High Cystatin C (≥0.82 mg/L) (*n* = 111)	*p*
Demographics				
Age, years	60.00 (52.00–67.00)	55.00 (48.00–60.00)	64.00 (55.00–70.00)	**<0.001**
Male gender, *n*	45 (22.5%)	13 (14.6%)	32 (28.8%)	**0.017**
Hypertension, *n*	50 (25.0%)	14 (15.7%)	36 (32.4%)	**0.007**
DM, *n*	24 (12.0%)	11 (12.4%)	13 (11.7%)	0.89
CKD, *n*	4 (2.0%)	0 (0.0%)	4 (3.6%)	0.070
Cardiovascular disease, *n*	14 (7.0%)	3 (3.4%)	11 (9.9%)	0.072
CVA, *n*	3 (1.5%)	2 (2.2%)	1 (0.9%)	0.44
HBV, *n*	25 (12.5%)	7 (7.9%)	18 (16.2%)	0.076
HCV, *n*	5 (2.5%)	1 (1.1%)	4 (3.6%)	0.26
Gout, *n*	7 (3.5%)	2 (2.2%)	5 (4.5%)	0.39
Autoimmune disease, *n*	2 (1.0%)	2 (2.2%)	0 (0.0%)	0.11
Metabolic syndrome, *n*	59 (29.5%)	27 (30.3%)	32 (28.8%)	0.82
Biochemical and physiological profiles				
SBP, mmHg	131.00 (119.00–141.00)	128.00 (117.00–140.00)	132.00 (121.00–143.00)	0.081
BMI, kg/m^2^	23.98 (22.23–26.35)	24.12 (21.29–26.25)	23.94 (22.43–26.67)	0.36
Overweight (BMI >24), *n*	99 (49.5%)	46 (51.7%)	53 (47.7%)	0.58
Central obesity, *n*	86 (43.0%)	33 (37.1%)	53 (47.7%)	0.13
Hgb, g/dL	13.70 (12.90–14.60)	13.70 (12.90–14.40)	13.70 (12.90–14.70)	0.54
Total cholesterol, mg/dL	208.50 (191.00–230.50)	207.00 (191.00–230.00)	210.00 (191.00–233.00)	0.92
LDL cholesterol, mg/dL	123.15 (105.85–146.10)	119.40 (102.90–146.60)	123.20 (109.00–145.90)	0.64
HDL cholesterol, mg/dL	56.55 (47.60–66.95)	56.50 (47.10–66.70)	56.60 (47.80–68.20)	0.79
Triglyceride, mg/dL	95.50 (69.00–139.00)	93.00 (67.00–155.00)	96.00 (70.00–132.00)	0.74
BUN, mg/dL	12.00 (10.00–15.00)	12.00 (9.00–14.00)	13.00 (11.00–16.00)	**0.004**
Creatinine, mg/dL	0.63 (0.55–0.76)	0.59 (0.54–0.66)	0.71 (0.57–0.82)	**<0.001**
eGFR, ml/min/1.73 m^2^	101.27 (87.33–115.08)	106.93 (97.66–123.31)	93.50 (78.15–109.51)	**<0.001**
Uric acid, mg/dL	5.10 (4.30–6.10)	4.70 (4.20–5.90)	5.30 (4.50–6.30)	**0.018**
Albumin, g/dL	4.70 (4.50–4.90)	4.80 (4.60–4.90)	4.60 (4.40–4.80)	**<0.001**
GPT, U/L	22.00 (17.00–29.50)	21.00 (16.00–30.00)	22.00 (18.00–28.00)	0.37
UACR, mg/g	5.75 (3.85–9.00)	5.90 (4.20–9.90)	5.60 (3.70–8.20)	0.21
Fasting glucose, mg/dL	96.00 (91.50–104.00)	96.00 (92.00–104.00)	96.00 (91.00–104.00)	0.93
HbA1C, %	5.60 (5.40–6.00)	5.60 (5.40–6.00)	5.70 (5.40–6.00)	0.32
Insulin, μIU/ml	5.80 (4.06–9.30)	5.74 (4.28–9.87)	5.95 (3.89–9.13)	0.96
HOMA-IR	1.43 (0.92–2.42)	1.41 (0.98–2.45)	1.46 (0.90–2.39)	1.00
HS-CRP, mg/L	0.94 (0.41–2.12)	0.72 (0.35–1.83)	1.05 (0.50–2.32)	0.093
Biomarkers				
Adiponectin, ng/ml	5.56 (3.31–10.14)	4.34 (2.57–8.26)	6.66 (3.93–10.60)	**0.002**
Leptin, ng/mL	12.00 (7.80–17.70)	12.60 (8.10–18.50)	11.10 (7.60–17.20)	0.35
Cystatin C, mg/L	0.86 (0.75–1.02)	0.73 (0.56–0.79)	1.00 (0.89–1.11)	**<0.001**
TNF-α, pg/mL	6.73 (5.70–8.17)	6.55 (5.18–7.78)	7.18 (5.73–8.59)	0.067
Total Vitamin D, ng/mL	24.29 (19.55–31.45)	23.20 (17.87–28.58)	25.71 (22.09–33.92)	**0.028**
Medication use				
OHAs, *n*	22 (11.1%)	10 (11.2%)	12 (11.0%)	0.96
Anti-hypertensives, *n*	46 (23.4%)	12 (13.5%)	34 (31.5%)	**0.003**
Painkillers, *n*	28 (14.8%)	14 (16.5%)	14 (13.5%)	0.56
**Outcome**				
RKFD, *n*	100 (50.0%)	65 (73.0%)	35 (31.5%)	**<0.001**

Abbreviations: BMI, body mass index; BP, blood pressure; BUN, blood urea nitrogen; CVA, cerebrovascular accident; DM, diabetes mellitus; eGFR, estimated glomerular filtration rate; HbA1C, glycated hemoglobin; GPT, glutamic pyruvic transaminase; HOMA-IR, homeostatic model assessment-insulin resistance; HDL, high-density lipoprotein; HS-CRP, high-sensitivity C-reactive protein; LDL, low-density lipoprotein; OHA, oral hypoglycemic agents; RKFD, rapid kidney function decline; TNF-α, tumor necrosis factor-α; UACR, urine albumin-to-creatinine ratio. Values in bold are statistically significant (*p* < 0.05).

**Table 4:** Variables showing prognostic significance for RKFD.

Parameter	Beta Coefficient	Standard Error	Odds Ratios (95% CI)	*p*-Value
**Univariable Analysis**				
Age, per 10 years	−0.18	0.14	0.84 (0.64, 1.09)	0.190
Male	−0.29	0.34	0.75 (0.38, 1.46)	0.398
Hypertension	0.43	0.33	1.54 (0.80, 2.93)	0.193
DM	0.58	0.45	1.78 (0.74, 4.29)	0.196
CKD	0.00	1.01	1.00 (0.14, 7.24)	1.000
Cardiovascular disease	0.31	0.56	1.36 (0.45, 4.08)	0.581
HBV	0.09	0.43	1.10 (0.47, 2.53)	0.831
HCV	−0.42	0.92	0.66 (0.11, 4.04)	0.653
Gout	0.95	0.85	2.58 (0.49, 13.62)	0.264
Metabolic syndrome	0.84	0.32	2.31 (1.23, 4.32)	**0.009**
Overweight (BMI > 24)	0.36	0.28	1.43 (0.82, 2.50)	0.204
Central obesity	0.41	0.29	1.51 (0.86, 2.64)	0.154
Hgb, per 1 g/dL	−0.05	0.10	0.95 (0.78, 1.17)	0.635
Total cholesterol, per 10 mg/dL	−0.11	0.04	0.90 (0.83, 0.98)	**0.011**
LDL cholesterol, per 10 mg/dL	−0.12	0.05	0.89 (0.81, 0.98)	**0.015**
HDL cholesterol, per 10 mg/dL	−0.15	0.10	0.86 (0.72, 1.04)	0.130
Triglyceride, per 10 mg/dL	0.03	0.02	1.03 (0.98, 1.07)	0.235
BUN, per 1 mg/dL	−0.05	0.04	0.95 (0.88, 1.03)	0.216
Creatinine, per 1 mg/dL	−2.76	0.98	0.06 (0.01, 0.44)	**0.005**
eGFR, per 10 mL/min/1.73 m^2^	0.26	0.07	1.29 (1.12, 1.49)	**<0.001**
Uric acid, per 1 mg/dL	−0.08	0.12	0.93 (0.74, 1.17)	0.514
Albumin, per 1 g/dL	0.51	0.54	1.66 (0.58, 4.78)	0.349
GPT, per 10 U/L	0.03	0.06	1.03 (0.91, 1.15)	0.676
UACR, per 1 mg/g	0.05	0.03	1.05 (0.99, 1.12)	0.093
Fasting glucose, per 10 mg/dL	0.13	0.07	1.14 (0.99, 1.31)	0.074
HbA1C, per 1%	0.34	0.20	1.40 (0.95, 2.07)	0.091
Insulin, per 10 μIU/mL	0.43	0.29	1.54 (0.87, 2.72)	0.140
Adiponectin, per 10 ng/mL	0.07	0.26	1.07 (0.64, 1.80)	0.800
Leptin, per 10 ng/mL	0.23	0.18	1.25 (0.88, 1.79)	0.216
HOMA-IR	0.14	0.10	1.15 (0.95, 1.39)	0.142
Cystatin C, low vs. high	1.77	0.31	5.88 (3.18, 10.89)	**<0.001**
HS-CRP, per 10 mg/L	−0.15	0.38	0.86 (0.41, 1.83)	0.698
OHA use	0.88	0.48	2.40 (0.93, 6.18)	0.069
Anti-hypertensive use	0.50	0.34	1.64 (0.84, 3.21)	0.145
Painkiller use	0.75	0.42	2.12 (0.92, 4.87)	0.078
Vegetarian	−0.06	0.37	0.94 (0.46, 1.96)	0.878
**Multivariable analysis**				
eGFR, per 10 mL/min/1.73 m^2^	0.34	0.14	1.40 (1.06, 1.85)	**0.018**
Cystatin C, low vs. high ^a^	3.01	0.59	20.35 (6.44, 64.29)	**<0.001**

^a^ Cystatin C < 0.82 mg/L was defined as low cystatin C; cystatin C ≥ 0.82 mg/L was defined as low cystatin C. Abbreviations: DM, diabetes mellitus; eGFR, estimated glomerular filtration rate; HbA1C, glycated hemoglobin; GPT, glutamic pyruvic transaminase; HOMA-IR, homeostatic model assessment-insulin resistance; HDL, high-density lipoprotein; HS-CRP, high-sensitivity C-reactive protein; LDL, low-density lipoprotein; OHA, oral hypoglycemic agents; RKFD, rapid kidney function decline; TNF-α, tumor necrosis factor-α; UACR, urine albumin-to-creatinine ratio. Values in bold are statistically significant (*p* < 0.05).

## Data Availability

The datasets used and/or analyzed during the current study are available from the corresponding author upon reasonable request.

## Supplementary Materials

The following supporting information can be downloaded at: https://www.mdpi.com/2227-9059/10/11/2789/s1, Figure S1: DCA plot to assess the clinical consequences of screening patients for the risk of RKFD using Cystatin C in addition to base covariates; Figure S2: Changes in eGFR according to the serum levels of cystatin C during the study; Table S1: Social psychology variables of the study population; Table S2: NRI and IDI analyses for the role of Cystatin C in stratifying individuals into high or low risk categories (re-classification); Table S3: Subgroup analysis of RKFD compared with low (<0.82 mg/L) and high levels (≥0.82 mg/L) of cystatin C; Table S4: Sensitivity analysis for risk estimation of cystatin C < 0.82 mg/L.

## References

[1] Eckardt K.-U., Coresh J., Devuyst O., Johnson R.J., Köttgen A., Levey A.S., Levin A. (2013). Evolving importance of kidney disease: From subspecialty to global health burden. Lancet.

[2] Ng J.K., Li P.K. (2018). Chronic kidney disease epidemic: How do we deal with it?. Nephrology.

[3] Alebiosu C.O. (2003). An update on ‘progression promoters’ in renal diseases. J. Natl. Med. Assoc..

[4] Chen T.K., Knicely D.H., Grams M.E. (2019). Chronic kidney disease diagnosis and management: A review. JAMA.

[5] Peralta C.A., Katz R., Sarnak M.J., Ix J., Fried L.F., De Boer I., Palmas W., Siscovick D., Levey A.S., Shlipak M.G. (2011). Cystatin C identifies chronic kidney disease patients at higher risk for complications. J. Am. Soc. Nephrol..

[6] Guebre-Egziabher F., Bernhard J., Funahashi T., Hadj-Aissa A., Fouque D. (2005). Adiponectin in chronic kidney disease is related more to metabolic disturbances than to decline in renal function. Nephrol. Dial. Transplant..

[7] Shankar A., Syamala S., Xiao J., Muntner P. (2012). Relationship between plasma leptin level and chronic kidney disease. Int. J. Nephrol..

[8] Lee B.T., Ahmed F.A., Hamm L.L., Teran F.J., Chen C.-S., Liu Y., Shah K., Rifai N., Batuman V., Simon E.E. (2015). Association of C-reactive protein, tumor necrosis factor-alpha, and interleukin-6 with chronic kidney disease. BMC Nephrol..

[9] Levin A., Li Y.C. (2005). Vitamin D and its analogues: Do they protect against cardiovascular disease in patients with kidney disease?. Kidney Int..

[10] Menon V., Shlipak M.G., Wang X., Coresh J., Greene T., Stevens L., Kusek J.W., Beck G.J., Collins A.J., Levey A.S. (2007). Cystatin C as a risk factor for outcomes in chronic kidney disease. Ann. Intern. Med..

[11] Wahba I.M., Mak R.H. (2007). Obesity and obesity-initiated metabolic syndrome: Mechanistic links to chronic kidney disease. Clin. J. Am. Soc. Nephrol..

[12] Zhang X., Lerman L.O. (2017). The metabolic syndrome and chronic kidney disease. Transl. Res..

[13] Navarro-García J.A., Fernández-Velasco M., Delgado C., Delgado J.F., Kuro-o M., Ruilope L.M., Ruiz-Hurtado G. (2018). PTH, vitamin D, and the FGF-23–klotho axis and heart: Going beyond the confines of nephrology. Eur. J. Clin. Investig..

[14] Blau J.E., Collins M.T. (2015). The PTH-Vitamin D-FGF23 axis. Rev. Endocr. Metab. Disord..

[15] Jhee J.H., Nam K.H., An S.Y., Cha M.-U., Lee M., Park S., Kim H., Yun H.-R., Kee Y.K., Park J.T. (2018). Severe vitamin D deficiency is a risk factor for renal hyperfiltration. Am. J. Clin. Nutr..

[16] Ostermann M., Zarbock A., Goldstein S., Kashani K., Macedo E., Murugan R., Bell M., Forni L., Guzzi L., Joannidis M. (2020). Recommendations on acute kidney injury biomarkers from the acute disease quality initiative consensus conference: A consensus statement. JAMA Netw. Open.

[17] Hsu C.-y., Yang W., Parikh R.V., Anderson A.H., Chen T.K., Cohen D.L., He J., Mohanty M.J., Lash J.P., Mills K.T. (2021). Race, genetic ancestry, and estimating kidney function in CKD. N. Engl. J. Med..

[18] Inker L.A., Eneanya N.D., Coresh J., Tighiouart H., Wang D., Sang Y., Crews D.C., Doria A., Estrella M.M., Froissart M. (2021). New creatinine-and cystatin C–based equations to estimate GFR without race. N. Engl. J. Med..

[19] Lee M.-J., Hsu H.-J., Wu I.-W., Sun C.-Y., Ting M.-K., Lee C.-C. (2019). Vitamin D deficiency in northern Taiwan: A community-based cohort study. BMC Public Health.

[20] Lipsy R.J. (2003). The National Cholesterol Education Program Adult Treatment Panel III guidelines. J. Manag. Care Pharm. JMCP.

[21] Matthews DR H.J. (1985). Rudenski AS, Naylor BA, Treacher DF, Turner RC, Homeostasis model assessment: Insulin resistance and beta-cell function from fasting plasma glucose and insulin concentrations in man. Diabetologia.

[22] Bonora E., Targher G., Alberiche M., Bonadonna R.C., Saggiani F., Zenere M.B., Monauni T., Muggeo M. (2000). Homeostasis model assessment closely mirrors the glucose clamp technique in the assessment of insulin sensitivity: Studies in subjects with various degrees of glucose tolerance and insulin sensitivity. Diabetes Care.

[23] Lin Y.F., Peng K.Y., Chang C.H., Hu Y.H., Wu V.C., Chung S.D. (2020). Changes in Glucose Metabolism after Adrenalectomy or Treatment with a Mineralocorticoid Receptor Antagonist for Primary Aldosteronism. Endocrinol. Metab..

[24] Liao P.-J., Ting M.-K., Wu I.-W., Chen S.-W., Yang N.-I., Hsu K.-H. (2021). Higher Leptin-to-Adiponectin Ratio Strengthens the Association Between Body Measurements and Occurrence of Type 2 Diabetes Mellitus. Front. Public Health.

[25] Chen L.-W., Kuo S.-F., Chen C.-H., Chien C.-H., Lin C.-L., Chien R.-N. (2018). A community-based study on the association between Helicobacter pylori Infection and obesity. Sci. Rep..

[26] Chu S.-C., Wang P.-H., Lu K.-Y., Ko C.-C., She Y.-H., Lee C.-C., Wu I.-W., Sun C.-Y., Hsu H.-J., Pan H.-C. (2022). Relationships Between Metabolic Body Composition Status and Rapid Kidney Function Decline in a Community-Based Population: A Prospective Observational Study. Front. Public Health.

[27] Young B.A., Katz R., Boulware L.E., Kestenbaum B., de Boer I.H., Wang W., Fülöp T., Bansal N., Robinson-Cohen C., Griswold M. (2016). Risk factors for rapid kidney function decline among African Americans: The Jackson Heart Study (JHS). Am. J. Kidney Dis..

[28] Pan H.-C., Huang T.-M., Sun C.-Y., Chou N.-K., Tsao C.-H., Yeh F.-Y., Lai T.-S., Chen Y.-M., Wu V.-C. (2022). Predialysis serum lactate levels could predict dialysis withdrawal in Type 1 cardiorenal syndrome patients. EClinicalMedicine.

[29] Pan H.-C., Huang T.T.-M., Huang C.-T., Sun C.-Y., Chen Y.-M., Wu V.-C. (2022). Urinary Biomarkers Can Predict Weaning From Acute Dialysis Therapy in Critically Ill Patients. Arch. Pathol. Lab. Med..

[30] Hin L., Lau T., Rogers M., Chang A. (1999). Dichotomization of continuous measurements using generalized additive modelling–application in predicting intrapartum caesarean delivery. Stat. Med..

[31] Chen H.-Y., Sun C.-Y., Lee C.-C., Wu I.-W., Chen Y.-C., Lin Y.-H., Fang W.-C., Pan H.-C. (2021). Ketoanalogue supplements reduce mortality in patients with pre-dialysis advanced diabetic kidney disease: A nationwide population-based study. Clin. Nutr..

[32] Funk M.J., Westreich D., Wiesen C., Stürmer T., Brookhart M.A., Davidian M. (2011). Doubly robust estimation of causal effects. Am. J. Epidemiol..

[33] Austin P.C., Stuart E.A. (2015). Moving towards best practice when using inverse probability of treatment weighting (IPTW) using the propensity score to estimate causal treatment effects in observational studies. Stat. Med..

[34] Rifkin D.E., Shlipak M.G., Katz R., Fried L.F., Siscovick D., Chonchol M., Newman A.B., Sarnak M.J. (2008). Rapid kidney function decline and mortality risk in older adults. Arch. Intern. Med..

[35] Koyner J.L., Bennett M.R., Worcester E.M., Ma Q., Raman J., Jeevanandam V., Kasza K.E., O’Connor M.F., Konczal D.J., Trevino S. (2008). Urinary cystatin C as an early biomarker of acute kidney injury following adult cardiothoracic surgery. Kidney Int..

[36] Shlipak M.G., Sarnak M.J., Katz R., Fried L.F., Seliger S.L., Newman A.B., Siscovick D.S., Stehman-Breen C. (2005). Cystatin C and the risk of death and cardiovascular events among elderly persons. N. Engl. J. Med..

[37] Buffet L., Ricchetti C. (2012). Chronic Kidney Disease and Hypertension: A Destructive Combination.

[38] Ficociello L.H., Perkins B.A., Roshan B., Weinberg J.M., Aschengrau A., Warram J.H., Krolewski A.S. (2009). Renal hyperfiltration and the development of microalbuminuria in type 1 diabetes. Diabetes Care.

[39] Palatini P. (2012). Glomerular hyperfiltration: A marker of early renal damage in pre-diabetes and pre-hypertension. Nephrol Dial Transpl..

[40] Palatini P., Dorigatti F., Saladini F., Benetti E., Mos L., Mazzer A., Zanata G., Garavelli G., Casiglia E. (2012). Factors associated with glomerular hyperfiltration in the early stage of hypertension. Am. J. Hypertens..

[41] Chagnac A., Herman M., Zingerman B., Erman A., Rozen-Zvi B., Hirsh J., Gafter U. (2008). Obesity-induced glomerular hyperfiltration: Its involvement in the pathogenesis of tubular sodium reabsorption. Nephrol. Dial. Transpl..

[42] Maeda I., Hayashi T., Sato K.K., Koh H., Harita N., Nakamura Y., Endo G., Kambe H., Fukuda K. (2011). Cigarette smoking and the association with glomerular hyperfiltration and proteinuria in healthy middle-aged men. Clin. J. Am. Soc. Nephrol..

[43] Hosokawa Y., Yamada Y., Obata Y., Baden M.Y., Saisho K., Ihara A., Yamamoto K., Katsuragi K., Matsuzawa Y. (2012). Relationship between serum cystatin C and serum adiponectin level in type 2 diabetic patients. Clin. Exp. Nephrol..

[44] Pittas A.G., Joseph N.A., Greenberg A.S. (2004). Adipocytokines and insulin resistance. J. Clin. Endocrinol. Metab..

[45] Vilela B.S., Vasques A.C.J., Cassani R.S.L., Forti A.C.E., Pareja J.C., Tambascia M.A., Investigators B., Geloneze B. (2016). The HOMA-Adiponectin (HOMA-AD) closely mirrors the HOMA-IR index in the screening of insulin resistance in the Brazilian Metabolic Syndrome Study (BRAMS). PLoS ONE.

[46] Dunkler D., Gao P., Lee S.F., Heinze G., Clase C.M., Tobe S., Teo K.K., Gerstein H., Mann J.F., Oberbauer R. (2015). Risk prediction for early CKD in type 2 diabetes. Clin. J. Am. Soc. Nephrol..

[47] Fang W.-C., Chou K.-M., Sun C.-Y., Lee C.-C., Wu I.-W., Chen Y.-C., Pan H.-C. (2020). Thermal Perception Abnormalities Can Predict Diabetic Kidney Disease in Type 2 Diabetes Mellitus Patients. Kidney Blood Press. Res..

[48] Atkins R.C. (2005). The epidemiology of chronic kidney disease. Kidney Int..

[49] Johnson R.J., Nakagawa T., Jalal D., Sánchez-Lozada L.G., Kang D.-H., Ritz E. (2013). Uric acid and chronic kidney disease: Which is chasing which?. Nephrol. Dial. Transplant..

[50] da Hora Passos R., Ramos J.G.R., Gobatto A., Mendonça E.J.B., Miranda E.A., Dutra F.R.D., Coelho M.F.R., Pedroza A.C., Batista P.B.P., Dutra M.M.D. (2016). Lactate clearance is associated with mortality in septic patients with acute kidney injury requiring continuous renal replacement therapy: A cohort study. Medicine.

[51] Ryoo S.M., Lee J., Lee Y.-S., Lee J.H., Lim K.S., Huh J.W., Hong S.-B., Lim C.-M., Koh Y., Kim W.Y. (2018). Lactate level versus lactate clearance for predicting mortality in patients with septic shock defined by sepsis-3. Crit. Care Med..

