# Supplementary material for: Serum Cystatin C Levels Could Predict Rapid Kidney Function Decline in A Community-Based Population
Source: Biomedicines. 2022 Nov 2;10(11):2789. doi: 10.3390/biomedicines10112789 (PMC9687581; doi:10.3390/biomedicines10112789)
Supplement: Supplementary file 1 [file biomedicines-10-02789-s001.zip › biomedicines-1983623-supplementary.pdf]

Supplementary Figure S1. DCA plot to assess the clinical consequences of screening patients for the risk of RKFD using Cystatin C in addition to base covariates. Y-axis is the net benefit of the decision strategy. Net benefit was defined as the net proportion of subjects with RKFD in whom a prediction model would provide benefit without applying a prediction model to subjects with good outcomes. For the subjects who did not develop RKFD (black line), forecasting with a combination of Cystatin C and base covariates did not yield a net benefit. When considering those who developed RKFD (grey), clear net benefits were seen for risk thresholds from 40 to 60%, forecasting that a combination of Cystatin C and base covariates (orange line) was beneficial. The net benefit was calculated as ((proportion of true positives) – (proportion of false positives))×pt/(1-pt), where pt is the threshold probability.

Abbreviations: DCA, decision curve analysis; RKFD, rapid kidney function decline

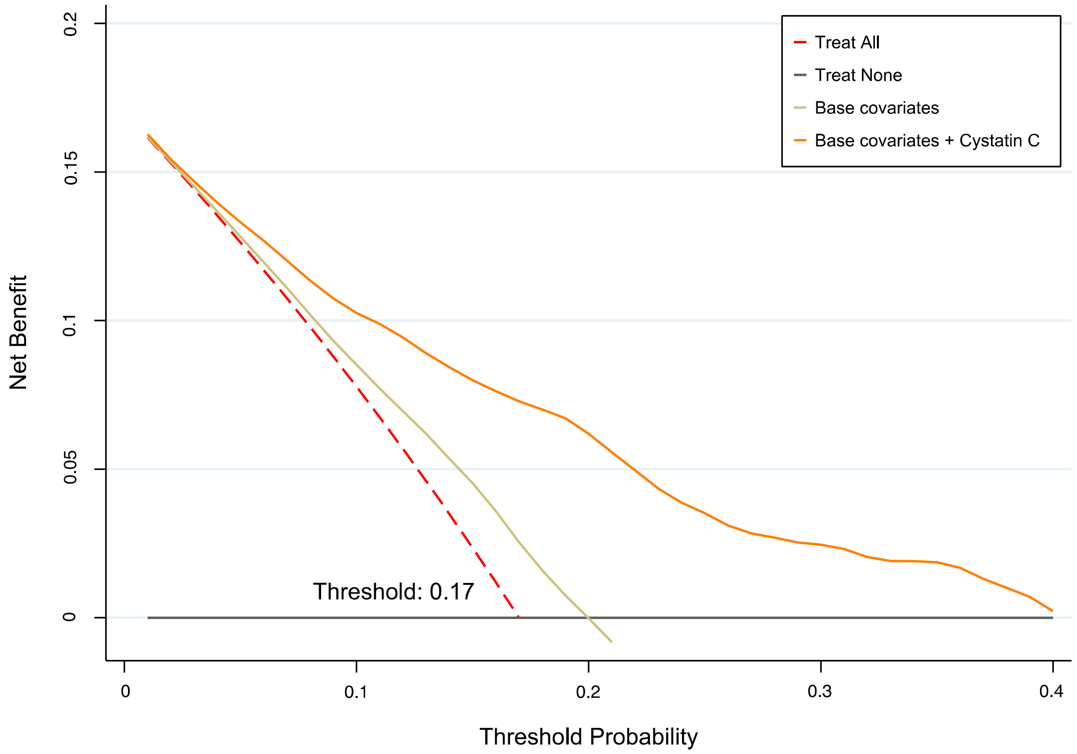

Supplementary Figure S2. Changes in eGFR according to the serum levels of cystatin C during the study

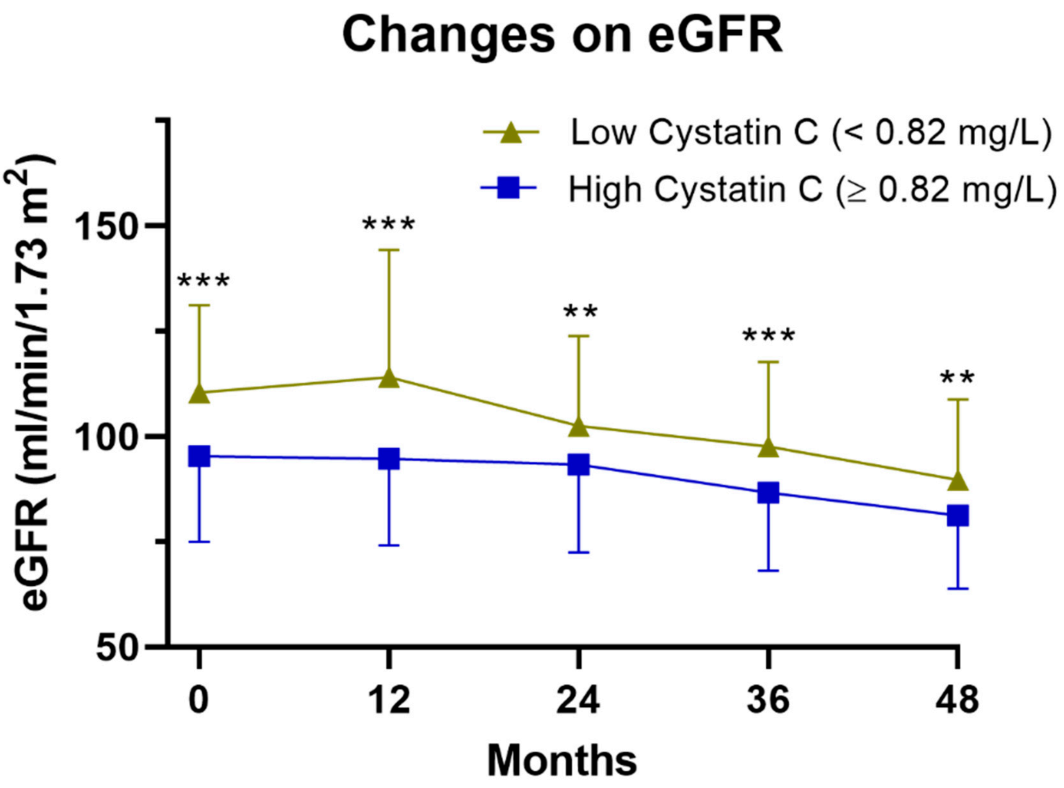

Abbreviations: eGFR, estimated glomerular filtration rate; RKFD, rapid kidney function decline; UACR, urine albumin-to-creatinine ratio

** P value < 0.01

*** P value < 0.001

Supplementary Table S1. Social psychology variables of the study population

	Total (n=200)	RKFD (n=100)	No RKFD (n=100)	*p* value
Education level				0.77
Illiterate, n	18 (9.1%)	7 (7.1%)	11 (11.2%)	
Elementary school, n	47 (23.9%)	27 (27.3%)	20 (20.4%)	
Junior high school, n	39 (19.8%)	20 (20.2%)	19 (19.4%)	
Senior high school, n	64 (32.5%)	30 (30.3%)	34 (34.7%)	
Undergraduate, n	26 (13.2%)	13 (13.1%)	13 (13.3%)	
Postgraduate, n	3 (1.5%)	2 (2.0%)	1 (1.0%)	
Substance use				
Smoking, n	23 (11.6%)	10 (10.1%)	13 (13.0%)	0.52
Betel nut, n	4 (2.0%)	3 (3.1%)	1 (1.0%)	0.31
Alcohol, n	37 (21.3%)	17 (19.1%)	20 (23.5%)	0.48
Dietary habits				0.88
Non-vegetarian, n	164 (82.4%)	82 (82.8%)	82 (82.0%)	
Vegetarian, n	35 (17.6%)	17 (17.2%)	18 (18.0%)	

Abbreviations: RKFD, rapid kidney function decline

Supplementary Table S2. NRI and IDI analyses for the role of Cystatin C in stratifying individuals into high or low risk categories (re-classification).

Model	Model with Cystatin C
Freq (Row percent)	reclassification(%)
Subjects without RKFD	
decreased probabilities	61
increased probabilities	39
Total	100
Subjects with RKFD	
decreased probabilities	29
increased probabilities	71
Total	100
Combined data	
Total	100

NRI(Categorical): 0.6571, *P*-value: 0.0001

Standard error: 0.1690

IDI: 0.26, *P*-value: < 0.001

Standard error: 0.031

Abbreviations: IDI, integrated discrimination improvement; NRI, net reclassification improvement.

Supplementary Table S3. Subgroup analysis of RKFD compared with low (< 0.82 mg/L) and high levels (≥ 0.82 mg/L) of cystatin C

	HR (95%CI)	p-value	Wald test(unweighted)
Age, years			**<0.001**
≤60	5.66 (2.56-12.51)	**<0.001**	
>60	2.08 (1.09-3.95)	**0.026**	
Gender			**<0.001**
Female	2.86 (1.78-4.60)	**<0.001**	
Male	3.43 (1.41-8.34)	**0.007**	
Hypertension			**<0.001**
no	3.48 (2.05-5.89)	**<0.001**	
yes	3.09 (1.45-6.55)	**0.003**	
Diabetes			**<0.001**
no	3.29 (2.09-5.19)	**<0.001**	
yes	1.43 (0.52-3.96)	0.491	
Metabolic syndrome			**<0.001**
no	3.46 (2.02-5.90)	**<0.001**	
yes	2.22 (1.15-4.26)	**0.017**	
Cardiovascular disease			**<0.001**
no	2.99 (1.93-4.63)	**<0.001**	
yes	7.63 (1.27-45.91)	**0.026**	
Gout			**<0.001**
no	3.07 (2.00-4.71)	**<0.001**	
yes	1.63 (0.27-9.85)	0.592	

Abbreviation: HR, hazard ratio

Values in bold are statistically significant ($p < 0.05$)

Supplementary Table S4. Sensitivity analysis for risk estimation of cystatin C < 0.82 mg/L.

	Unadjusted		Adjusted	
	HR (95%CI)	p-value	HR (95%CI)	p-value
Different PS modeling				
With IPTW (n= 200)	2.61 (1.76-3.87)	**<0.001**	5.65 (2.69-11.88)	**<0.001**
With stratification (n= 200)	2.42 (1.57-3.71)	**<0.001**	5.54 (2.62-11.73)	**<0.001**
With overlap weighting (n= 200)	2.53 (1.70-3.75)	**<0.001**	5.53 (2.65-11.53)	**<0.001**
1:1 PSM (n= 144)	2.63 (1.68-4.13)	**<0.001**	10.32 (3.25-32.74)	**<0.001**

Abbreviations: HR, hazard ratio; IPTW, inverse probability of treatment weighting; PS, propensity score; PSM propensity score matching

Values in bold are statistically significant ($p < 0.05$)
